# *In situ* size sorting in CVD synthesis of Si microspheres

**DOI:** 10.1038/srep38719

**Published:** 2016-12-08

**Authors:** M. Garín, R. Fenollosa, L. Kowalski

**Affiliations:** 1 Grup de recerca en Micro i Nanotecnologies, Departament d′Enginyeria Electrònica, Universitat Politècnica de Catalunya, c/Jordi Girona Pascual 1–3, Barcelona 08034, Spain; 2 Instituto de Tecnología Química (CSIC – UPV), Universitat Politècnica de Valencia, Av. Tarongers s/n 46022, Valencia, Spain

## Abstract

Silicon microspheres produced in gas-phase by hot-wall CVD offer unique quality in terms of sphericity, surface smoothness, and size. However, the spheres produced are polydisperse in size, which typically range from 0.5 μm to 5 μm. In this work we show through experiments and calculations that thermophoretic forces arising from strong temperature gradients inside the reactor volume effectively sort the particles in size along the reactor. These temperature gradients are shown to be produced by a convective gas flow. The results prove that it is possible to select the particle size by collecting them in a particular reactor region, opening new possibilities towards the production by CVD of size-controlled high-quality silicon microspheres.

Silicon microspheres constitute a promising platform for developing experiments and applications in different technological areas such as metamaterials, photonics and optoelectronics, to name a few^1^,^2^,^3^,^4^,^5^. Currently, there are several approaches for obtaining such particles in the micrometer and submicrometer range, namely high-temperature trisilane thermolysis in supercritical solvents^6^,^7^, Chemical Vapor Deposition (CVD) with disilane as precursor^8^,^9^, laser ablation^10^,^11^,^12^ and mechanical milling plus laser irradiation^13^. Each method has advantages and drawbacks in terms of purity, spherical perfection, and size dispersion of the particles. For instance, in the case of laser ablation particles with high purity, although not a perfect spherical condition, have been obtained. In the case of trisilane thermolysis, the degree of control in size and dispersion should be emphasized; however, the obtained particles still suffered from a lack purity probably because of the solvent environment where they were synthesized. Of course, any of them may be suitable depending on the application requirements.

In the CVD route based on disilane^8^,^9^ particles nucleate and growth in gas-phase, yielding particles with very good spherical perfection, surface smoothness and material purity. These are important features specially in the field of optoelectronics because they enable the particles to behave as both proper semiconductors and high-quality-factor Mie resonators. For instance, photodiodes based on single silicon microspheres produced in this way were recently realized^4^, which were able to trap and detect light in the near infrared at the silicon’s band gap edge thanks to the resonant effect. In spite of the high-quality of the particles produced, the CVD method presents almost no control of the particle size. A typical particle distribution shows a modal size between 1–1.5 μm with a standard deviation of 35% or higher, although particles down to 0.5 μm and up to 5 μm can be found with ease. There are several applications that benefit from a disperse distribution of silicon resonators, such as sunlight harvesting^14^ or as UV/VIS/IR filter for cosmetics^15^. However, many other applications require a precise control over both the size and the dispersion of the particles. For instance, self-assembled colloidal crystals^1^,^16^ and metasurfaces^17^ require a dispersion below 3% for avoiding crystal defects while the particle size and geometry define the working wavelength range. Also, silicon microspherical resonators can be used as building blocks in coupled-cavity devices such as chain waveguides^18^,^19^,^20^, delay lines^21^, channel add-drop filters^22^, high-resolution spectroscopy^23^, lasers^24^,^25^, and optical memories^26^,^27^ to name a few. These devices are even more stringent in the size dispersion, since they would rely in microspheres with identical, or very well defined, high-*Q* optical resonances. Still, there are applications for high-index dielectric microspheres, such as super-resolution optical microscopy^28^,^29^,^30^, that are less stringent in terms of particle size distribution.

It is therefore highly desirable to achieve size and dispersion control with the CVD method. However, it constitutes a challenging goal because particles grow in an aerosol process where several phenomena occur at the same time, namely nucleation, coalescence, convection, and settlement by gravity^31^,^32^,^33^. In this complex scenario of chemical reactions and physical forces we have found that, under several conditions which include reactor geometry and heating strategy, two important processes related with particle size sorting can be realized. The first one consists of preventing particles with diameter below a certain value from depositing onto the supporting substrate, the second one is a particle size sorting effect where microspheres are deposited in different substrate zones depending on their diameter. These effects produce areas containing well separated microspheres. Therefore they can keep their optical resonances without being influenced by other neighbors, or by smaller undesirable particles that act as defects when deposited on the bigger ones. Here we provide a quantitative study of these effects and we give a qualitative explanation based on the equilibrium between convection, gravity, and thermophoretic forces.

## Sphere synthesis

**Figure 1:**
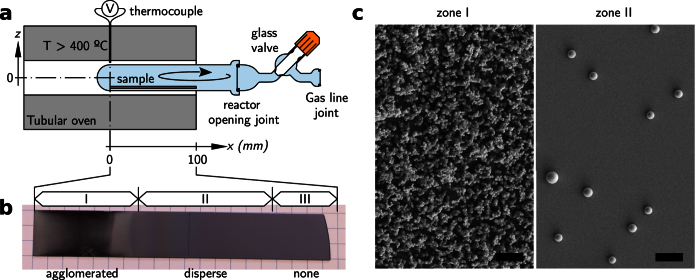
Synthesis of silicon microspheres by CVD. (**a**) Schematic of the hot-wall CVD process configuration. (**b**) Photography of a typical as-synthesized sample over a 5 mm grid paper with three indicated regions containing agglomerated particles (region I), disperse particles (region II) and no deposited particles (region III). (**c**) SEM images, same magnification, of synthesized spheres as deposited over the sample in regions I and II. Both scale bars are 10 μm.

The synthesis by CVD of silicon microspheres has been presented elsewhere^8^; the following is a description of the procedure and the main experimental observations concerning the present study. Silicon microspheres are synthesized by thermal decomposition of pure disilane gas (Si_2_H_6_) in a closed reactor and at temperatures above 400 °C. This has been realized so far in a bare quartz tube by introducing part of it in a tubular oven. This is a key point for achieving an appropriate temperature gradient and gas convection condition and, as a result, for achieving the aforementioned effects of particle size sorting. In this work we have used as a reactor a 300 mm long quartz tube with an internal diameter of 25 mm. Figure 1a shows the schematic of the set up. The reaction starts by partially inserting the reactor in horizontal position into the tubular oven at 420 °C. After a while, typically several minutes, particles start to nucleate and grow in the gas-phase, eventually falling by gravity onto any substrate previously introduced inside it. The fact that the reactor is half inserted into the oven creates a strong horizontal thermal gradient, where the tip of the reactor (in the center of the oven) is at the set point temperature while half of it is exposed to room temperature conditions (see Fig. 1a). This gradient originates a convective gas movement that carries silicon particles along the reactor. In fact, this convective movement is visible to the naked eye under proper lighting (*e.g.* with a laser pointer) thanks to light scattering effects by the thin mist of floating particles. As the reaction goes on, the precursor gas gets exhausted and the synthesis eventually decays. Finally, the exhaust gases are extracted and the sample is retrieved.

Figure 1b,c show a typical sample obtained by this method, where three main zones can be distinguished. The left region (zone I) is a layer of agglomerated spherical particles, poly-disperse in size, forming a sponge-like structure that appears as dark gray color. Notice, however, that the leftmost region, the first few millimeters of the sample, present a much lower density of particles. The center region of the sample (zone II) presents well isolated microspheres with relatively uniform sizes, which, in essence, is the leitmotiv of this study. Finally, in the rightmost part of the sample (zone III) there is no particle deposition. As a first approximation, we expect that the orography should reflect the physico-chemical processes occurring in the gas volume right above any sample region. As a result, the high amount of deposited particles found in zone I should come from the higher nucleation rate occurring owing to the higher temperatures at that particular region of the reactor. Those higher temperatures should also favor the coalescence of particles, cluster de-hydrogenation, and conformal growing^34^,^35^,^36^,^37^,^38^. Furthermore, not only should the particles reach the substrate by gravity but also driven by diffusion due to such high nucleation rate. The abrupt frontier between zone I and II, in our case occurring at a temperature around 400 °C for a pressure of 0.1 bar, indicates the strong sensitivity to temperature of the nucleation process. Such an effect has been reported elsewhere^39^,^40^ for silane decomposition. Regarding zone II, because of the lower gas temperature around this region, nucleation and coalescence are much less probable to occur. Therefore, other processes concomitant of gas convection are expected to manifest here in a more pronounced way, which will be fully discussed in the next sections.

## Particle characterization

In order to achieve a more detailed picture of the outcome from the CVD process, we have quantified by imaging techniques the density and size distribution of particles over the full surface of the 15 × 100 mm sample shown in Fig. 1b. In particular, since the variation occurs mainly through the axis of the reactor, we have taken SEM images every 5–10 mm along this axis. However, for averaging purposes, we have taken 75 equidistant images (every 200 μm) along the perpendicular axis. Images are taken at different magnifications depending on the average size and density of the particles. Regions with sparse big spheres are acquired at lower magnifications, so as to catch more spheres by image, while regions with dense small particles are acquired at higher magnifications, so that the particles can be correctly identified and measured. The number and size of the particles was determined with sub-pixel accuracy using a Hough-transform-based algorithm. This method is very robust and has a very high detection success rate even for partially overlapped or lightly aggregated particles. With this method we can accurately estimate both the particle density and distribution in thin layers of particles, with none or occasional aggregation. However, for thick layers it can only be used to estimate the topmost particle distribution, since there is no direct view of the particles underneath and the success rate decreases. It must be emphasized that the sample studied was synthesized at a relatively low pressure, 0.1 atm, so that the sponge-like layer occurring in region I was relatively thin. In fact, the surface of the substrate is still visible through the sponge openings.

**Figure 2:**
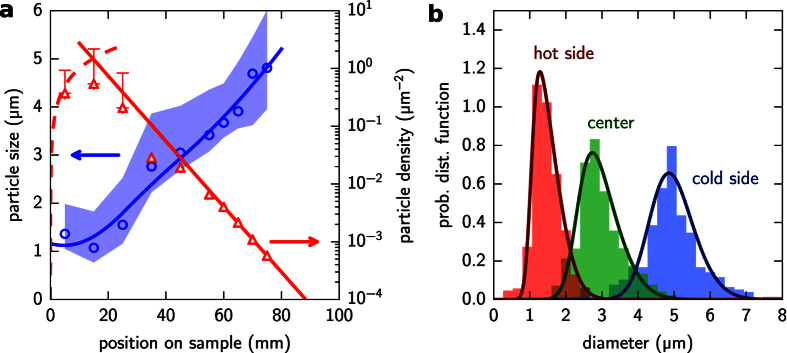
(**a**) Particle size distribution (circles) and particle density (triangles) as a function of the sample position. The colored band represents the 90% interval of the size distribution. The lines are added solely for eye-guiding purposes. (**b**) Distribution of particle size at three different sample locations. Solid lines represent skew-normal fittings.

The first thing we notice in the results, Fig. 2, is the strong dependence of the particle density along the reactor axis, which is a natural consequence of the particle transport processes from zone I where nucleation occurs. As Fig. 2a shows, the particle density decays exponentially as we move away from the hot side, except at the first 10–15 mm of the sample, that show the opposite behavior. In fact, the decrease is drastic and seems to go to nearly zero at the hot edge of the sample, which is clearly observable to the naked eye (see Fig. 1b). It is worth mentioning that at the highest density region, distances around 5–25 mm from the oven’s center, the particle density is underestimated due to the large aggregation of particles by up to a factor 3 in the worst case. We have roughly estimated this deviation based on manual counting of particles in a few images, leading to the error bars in Fig. 2a.

The second trend we observe in Fig. 2a is the pronounced change of the average particle size along the reactor axis. The modal particle size increases from around 1 μm in the beginning (hot side of the reactor), to nearly 5 μm at a distance of 75 mm. This variation is quite linear, except for the first 10–15 mm of the sample. The shaded region in Fig. 2a represents the 90% distribution interval, where it can be clearly seen that the total size span is much wider than the particle dispersion at any position. The size distribution, Fig. 2b, tends to resemble a skewed Gaussian distribution near the reactor start (hot, oven’s center), whereas it becomes more symmetrical towards the reactor opening. Also, the standard deviation of the diameters tends to reduce as we move along the reactor from around 30%, in the hot side, to around 10% in the cold side. This is noticeably lower than the overall dispersion, taking into account all of the microspheres, which is close to 35%.

**Figure 3:**
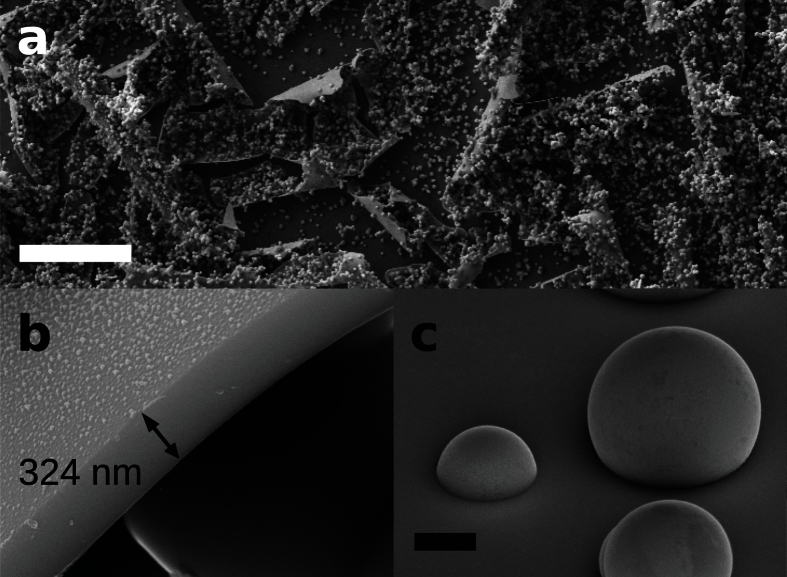
(**a**) Particle synthesis over a quartz sample; the scalebar represents 50 μm. The stress peals off the grown a-Si:H layer. (**b**) Detail of (**a**), where the thickness of the layer can be estimated. (**c**) Close view of spheres synthesized over a silicon substrate at the hot reactor end; the scale bar represent 500 nm.

In addition to the particle size and quantity distributions, the careful analysis through imaging of the as-deposited particles also confirms some characteristics of the particle synthesis. During the process, a thin conformal layer of silicon is formed in addition to the spherical particles. This layer clearly forms at the hottest part of the reactor, up to around 50 mm from the reactor’s hot end, creating a thick gray layer covering the reactor walls. This CVD layer tends to peel off when using quartz substrates instead of bare silicon ones (Fig. 3a), what makes easy to see that this layer can reach values up to few hundreds nanometers (Fig. 3b). Furthermore, when looking at the particles near the hottest reactor point, it can be observed that they appear partially buried at the surface (Fig. 3c). This reveals that the conformal CVD layer grew around 100–300 nm at that point after some particles had already been deposited on the surface, what clearly indicates that conformal growth can contribute significantly to the final particle size. Further away, into the colder regions, this layer becomes much thinner and seems to not contribute significantly to the particle size but to create just a contact point between substrate and particle.

**Figure 4:**
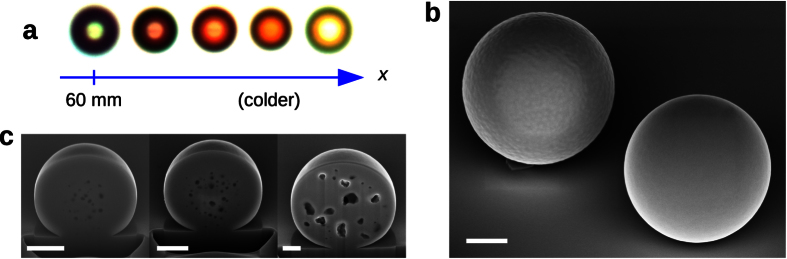
(**a**) Optical microscope images of amorphous microporous spheres showing photoluminescence found at different reactor positions. The images were in overexposure conditions in order to enhance the colors. (**b**) SEM image, in-lens detector, of two nearby amorphous microspheres with different texture and contrast. (**c**) Cross section of several recrystallized spheres obtained by carving with a focused ion beam facility. Scale bars are 1 μm.

At distances beyond 50 mm, away from the reactor’s end (the hottest point), the microspheres also start to show diverse signs of increasing porosity. For instance, a small fraction of the microspheres, say less than 1%, tend to have a reddish color under examination by optical microscopy (specially under overexposure conditions), instead of their typical dark color. As we move further away, such particular spheres shift from a red tone towards a green/yellow one (Fig. 4a). These colored microspheres are not representative of the whole set since they are produced in very small amounts. However, they provide hints about the existence of porosity because such colors are associated to the photoluminescence (PL) of porous silicon, and it is well known that the PL shifts towards shorter wavelengths as the porosity increases. Also, under SEM inspection, some particles in this region also show a characteristic rough surface and large charging artifacts (Fig. 4b), which we believe are also indications of a particularly high porosity. It is worth mentioning that we have previously confirmed PL in porous silicon microspheres obtained by partially frustrating the CVD decomposition reaction, *i.e.* by using short reaction times^9^. In that work, porous spheres were produced in large amounts everywhere in the reactor and exhibited very distinct and bright colors, in contrast to the dull colors and the low amount of porous spheres found in this work. In any case, the existence of some amount of inner porosity influencing the resonating modes in silicon microspheres was recently confirmed after recrystallizing the spheres through thermal annealing^41^, since small bubbles form into the sphere volume that can be revealed by carving it with a focused-ion-beam system (see Fig. 4c). It turns out that most particles in the cold region show a certain degree of porosity even if they do not become luminescent or rough.

All the above observations suggest that the different processes involved in the microsphere formation can work at very different speeds. First, the microspheres grow rapidly by coalescence of smaller clusters, with a high content of hydrogen that comes from the precursor gas. At the same time, the microspheres dehydrogenate, substituting hydrogen by silicon, further growing by conformal silicon growth. The balance between such processes depends on the temperature, and therefore the reactor position, dehydrogenation and conformal growing being much slower in the colder regions. Consequently, the observed increase in porosity in region II would be related to this effect.

## Gas convection simulation

**Figure 5:**
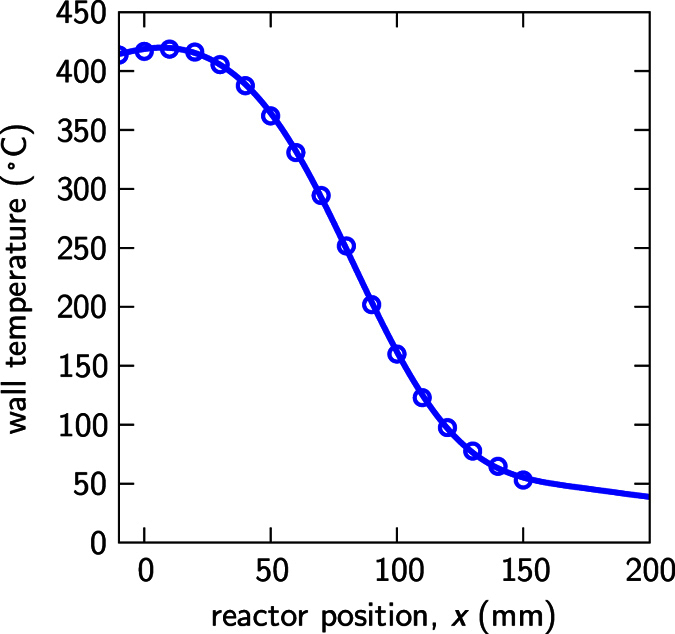
Measured reactor’s wall temperature profile. Position *x* = 0 mm corresponds to the hot end of the sample and position *x* = 100 mm corresponds to the oven’s entrance.

Gas convection is a key point among the complex physico-chemical processes for understanding the obtained particle size distributions, since it is responsible of the transport of particles through large distances and of inducing strong temperature gradients in the gas volume as we show below. As a matter of fact, we know from past experience that, in reactors where convection is somehow impeded, poly-disperse silicon microspheres are still obtained but no such particle size sorting effects are observed. Those reactors include set-ups similar to that of Fig. 1a but with smaller diameters, say for instance 15 mm, and capsule-like reactors with volumes in the mm^3^ range that are placed at the center of the oven where temperature gradients and therefore convection are vanishing. In order to get a deeper insight into the convection phenomenon, we have simulated the temperature and gas speed distributions inside the reactor by a finite element method using a commercial multiphysics package combining fluid dynamics with thermal behavior under gravitational volumetric force conditions. The reactor was modeled as a cylinder 260 mm in length, 25 mm in internal diameter, and with spherical caps on both ends, which is a very good approximation specially at the side where the reaction takes place. The sample was not considered in the simulation since it lays flat and close to the bottom of the reactor. As boundary conditions we fixed the reactor walls’ temperature (cylindrical symmetric) following a temperature profile along the reactor axis that was experimentally measured (Fig. 5). Since we work in continuum flow, the no-slip conditions, *i.e.* zero velocity, were applied to all internal reactor walls. Finally, all calculations were performed under laminar flow regime, assumption that is supported by the experimental observation of the particles flow inside the reactor, which is steady and smooth. Also, for simplicity we assumed that the gas is pure Si_2_H_6_ with the standard room temperature parameters, neglecting gas change during decomposition.

**Figure 6:**
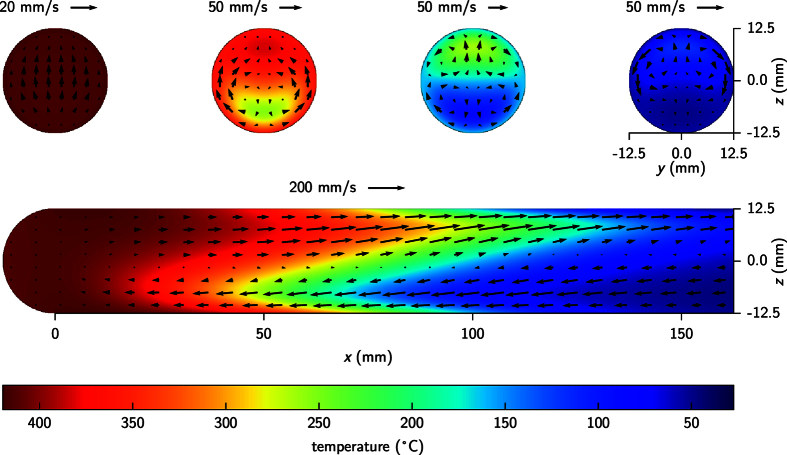
Calculated temperature distribution at different planes inside the reactor. The arrows show the gas velocity field (plane projection) due to convection. Notice that the velocity scale is different in each panel.

**Figure 7:**
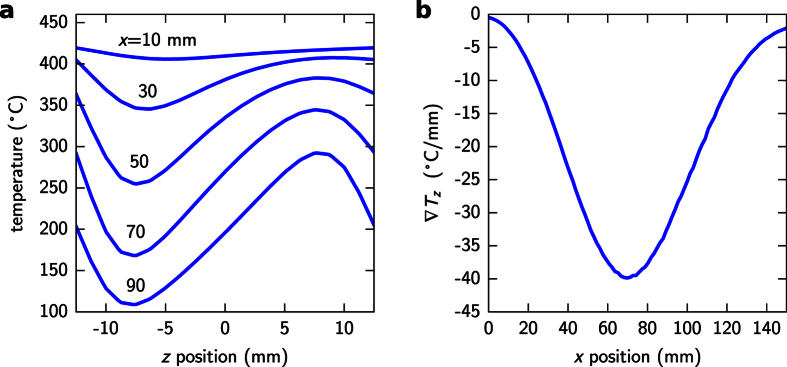
(**a**) Temperature of the gas along the z direction at different reactor positions in the *x* direction. (**b**) Temperature gradient, *z* component, along the reactor at 100 μm above the reactor’s bottom wall (*z* = −12.4 mm).

Figure 6 shows the gas temperature (colors) and gas velocity (arrows) distribution at several planes. The lower diagram corresponds to the long reactor symmetrical plane, defined by the *x* (horizontal) and *z* (vertical) coordinates, and the upper diagrams correspond to four cross sections of the reactor, along *yz* directions, at different longitudinal (*x*) positions, namely 0, 50, 100, and 150 mm. As expected, because of the high temperature difference between both ends of the reactor, there is a strong convection process along the *xz* plane, where hot gas located at the upper parts moves towards the cold end of the reactor and returns once it has been cooled down to the opposite hot end through the lower parts. The maximum gas speed in this plane occurs at the oven entrance (*x* = 100 mm) and it reaches values around 150 mm/s. The cross sectional planes show complex convection patterns depending on their *x* position. For instance, the gas at plane *x* = 0 mm moves completely upwards, whereas the gas moves upwards and downwards within the same *yz* plane at colder locations. Nevertheless, the speed of the gas in these transverse planes is in general one order of magnitude smaller than that at the *xz* plane. Also, the gas speed tends to vanish near the reactor walls due to no-slip boundary conditions due to the continuum flow regime and, particularly, the component perpendicular to the surface due to the laminar flow regime. On the other hand, the diagrams do show strong temperature gradients in the vertical direction and at intermediate longitudinal positions (50 < *x* < 100 mm), which are produced by the natural convection of gas: hot gas moving near the top and cold gas moving near the bottom. For the sake of convenience, the temperature variation along the *z* direction at different *x* locations, and the vertical temperature gradient along the *x* direction at z = −12.4 mm (100 μm above the reactor bottom wall) has been plotted in Fig. 7a,b, respectively. These temperature gradients, that can reach values up to 40 °C/mm near the bottom of the reactor wall, play a key role in the particle size sorting effects because they induce remarkable thermophoretic forces that tend to push particles away from the surface. This is a well known phenomenon in the field of aerosols^42^. In particular, the appearance of a floating dust onto a heated substrate was reported a long time ago in experiments of gas-phase decomposition of silane in horizontal epitaxial reactors^43^.

## Discussion

The observed effects of particle size sorting could be understood by considering two opposite forces of comparable magnitude that act on the particles at the same time along the vertical direction. These are the gravitational force *f*_g_ that makes the particles tend to deposit onto the substrate and the thermophoretic force *f*_th_, which arises from the strong temperature gradients at the bottom of the reactor (Fig. 7b) and pushes the particles upwards. Although the gas flow produces a strong drag force along the reactor, the *z* component of the gas speed is negligible near the bottom of the reactor. This is reasonably true within the first 100 micrometers from the bottom of the reactor up, which is a distance much bigger than the particle size. Therefore, the main role of the convection here consists of providing a strong temperature gradient at the bottom of the reactor, rather than a force.

The key point of the particle size sorting effect is the fact that both forces, gravitational and thermophoretic, are comparable in magnitude but depend on the particle size in a different way. The gravitational force inside the gas is determined with the common expression


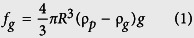


where *R* is the radius of the spherical particle, 4/3π*R*^3^ is the particle volume, ρ_p_ and ρ_g_ are respectively the volumetric mass density of the particle (silicon) and the gas at the working pressure and temperature, and *g* is the Earth’s gravity. On the other hand, the thermophoretic force can be calculated by using the following interpolation formula^44^, which is valid over the entire Knudsen number range: 0 < λ/*R* < ∞


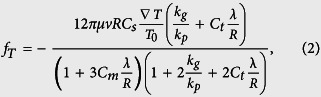


where λ is the mean free path, Δ*T* and *T*_0_ are respectively the temperature gradient and the temperature value at the particle position, μ is the gas viscosity, ***v*** equals to the ratio μ/ρ_g_, *k*_p_ and *k*_g_ are the thermal conductivities of the particle and the gas, respectively, and *C*_m_, *C*_s_ and *C*_t_ are numerical factors from kinetic theory. Table 1 summarizes the values of all the parameters used in this calculation. The Knudsen number is from about 0.09 to 0.46 for particle diameters from 5 to 1 micrometer respectively.

**Figure 8:**
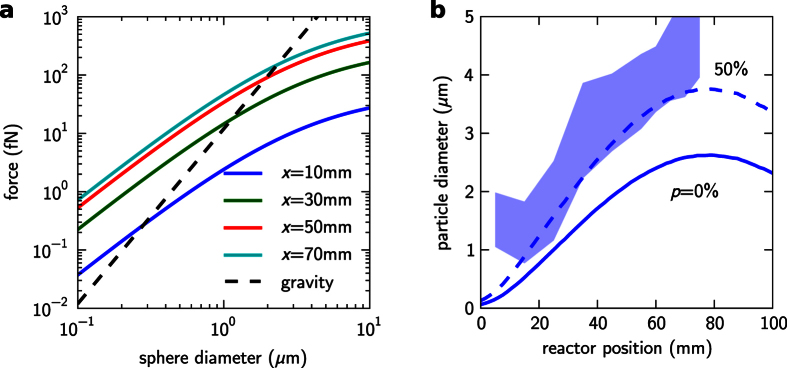
(**a**) Comparison of the gravitational force (dashes) and the vertical component of the thermophoretic force (colors) as a function of the particle diameter and at different locations of the reactor. Notice that the gravitational force and the vertical component of the thermophoretic force are parallel but in opposite directions. (**b**) Calculated minimum particle size that can be deposited over the sample considering solid particles (line) and particles with a 50% porosity (dashes). For comparison purposes, the colored band represents the 90% interval of the experimental size distribution.

Figure 8a shows the variation of the gravity force (dashed black line) and the thermophoretic force (solid lines) with the diameter of the particle. Here, the particle has been considered as a perfect sphere made of pure silicon, from which its density and volume can be determined. Because the temperature gradient is strongly dependent on the position along the reactor, the thermophoretic force was calculated at different locations in *x*. In all these cases we chose a *z* position of 100 micrometers, from the bottom of the reactor, for determining the temperature and its gradient. The point at which the gravity force equals to the thermophoretic force at any *x* position defines the minimum size of particle that will be deposited at that location assuming there are no other influencing factors. No smaller particles can be deposited there because they would be repelled off the surface by the thermophoretic force. In the case of *x* = 10 mm (dark blue curve) such crossing point occurs at a sphere diameter of about 300 nm. However, because the temperature gradients get more pronounced as *x* increases (Fig. 7b), the thermophoretic force and therefore the minimum diameter (*d*_min_ from now on) also increases at colder locations.

The curve in Fig. 8b (solid line) represents the calculated minimum particle size along the reactor, which seems to agree qualitatively with the experimental results (shaded region), although the computed values are sensibly lower especially at the colder reactor regions. There are, however, other aspects of the process that must be taken into account to understand the experimental particle distribution. First of all, as discussed in section 3, there are strong evidences suggesting that particles are highly porous in the initial stages of its formation due to a rapid growth through coalescence of nanoparticles in the gas phase. As a result, we must assume that spheres can still be porous when they make contact with the surface, specially in the colder reactor side (*x* > 50 mm). This would considerably lower the gravitational force while the thermophoretic force is unaffected, raising the diameter of the deposited particles. As a matter of fact, considering a particle porosity of 50% at the moment of the deposition (dashed line in Fig. 8b) would better match the experimental distribution registered along most of the reactor. The particle porosity, however, does not seem to explain the minimum particle size of around 1 μm near the beginning of the reactor (*x* < 20 mm), which should decrease to nearly zero according to the calculations. On the other hand, this can be explained by the much thicker conformal CVD layer growth at this region. We estimate that this layer can achieve several hundreds of nanometers in the thickest point, what would account for an extra particle diameter in the range 0.5–1 μm. This effect alone would perfectly account for the observed differences. At the rest of the reactor (x > 20 mm), the contribution of this CVD conformal layer to the total diameter is negligible and the role of this process seems to be to solidify and smooth the sphere’s surface.

Besides the existence of a minimum diameter as deduced from the balance between the gravitational and the thermophoretic forces, it is worth mentioning that the maximum size of the particles is also limited. However, the maximum achievable sphere diameter should be more related to the geometrical constrains of the reactor, because once a sphere has reached a certain value so that the gravitational force acting on it becomes larger than the thermophoretic force, it deposits onto the substrate and it can not continue growing either by coalescence or by any other mechanism like conformal growing (in zone II).

Although convection and temperature gradients seem to explain the particle sorting effects, the presented work does not offer a full explanation with regard of the particle density distribution. As already mentioned, the exponentially decaying distribution seems compatible with a particle transport from the high nucleation region at the hottest reactor side. However, this trend is entangled with the size distribution of spheres and, therefore, it could be also correlated to the dynamics of nanoparticle coalescence and particle growth. On the contrary, the sudden lowering on the particle density near the edge of the sample, *x* = 0, approaching zero, can be explained in terms of the convective gas flow at that point. At the edge of the sample there is an upright convective flow since this is the hottest point of the reactor (see upper left panel in Fig. 6). Therefore, at this point the particles are expected to be lifted by the gas flow avoiding its deposition near the end of the sample.

Finally, regarding the size dispersion, although a reduction in the standard deviation of diameters down to values of 10% was obtained, this is still far from values of 1–2%, which are usually considered as mono-disperse, and that are necessary for developing those applications mentioned in the introduction. Such mono-disperse distributions can be obtained in liquid solutions for silica and polystyrene microspheres by using the Stöber method^45^, and in supercritical solvents for silicon microspheres^6^,^7^. Aerosol processes are in principle more complicated and in the case of the CVD route with di-silane as a precursor gas there are still many aspects that remain unknown. For example, how particle synthesis and aggregation occurs at different reactor regions, the relative significance of drag and diffusion as particle transport mechanisms, and how the dynamic gas conditions in the closed reactor ambient affects the reaction through time. These are important questions that need to be further investigated, and require perhaps more complex numerical models and sophisticated experiments, which are beyond the aim of this paper. They make it difficult to envisage the limits of this technology regarding the goal of mono-dispersity. However, there is still room for improvement. On one hand, strategies can be developed for simplifying the aerosol synthesis process. For instance, it has been shown that particle dispersions of near 5% can be obtained by reducing coalescence in a CVD process with silane as a precursor gas^46^, although the diameter of such particles was in the range of tens of nanometers. On the other hand, additional size sorting elements could be used during the synthesis process itself or afterwards. They include the usage of differential mobility analyzers^47^ and even more recent and sophisticated techniques^48^,^49^,^50^ which take advantage of the optical resonant forces occurring in the particles, and that can in principle provide very high size accuracies in the order of 1/*Q* where *Q* corresponds to the quality factor of the resonating mode. This method based on the spectral-selective resonant propulsion of the microspheres could be used in vacuum, gas and liquid environments, and in combination with more common techniques, such as sedimentation, centrifugation and separation by membranes that could provide a first reduction of the dispersion before undertaking the more fine sorting process. However, these methods require a production yield much higher than the one obtained in our closed reactor system, which is quite limited. One possibility of attaining both efficient size sorting and reasonable throughput is the development a continuous CVD system, which currently constitutes a research goal.

## Conclusions

In summary, we have presented a detailed analysis of the particle density and size distribution of silicon microspheres produced by hot-wall CVD in a closed reactor. The results have revealed the existence of a natural particle sorting effect that allows to control the desired particle size by just selecting the appropriate region where particles are collected. Furthermore, the particle dispersion has been reduced from a starting 35%, for the overall particle production, down to ~10% at certain reactor regions. Theoretical calculations of the temperature distribution and gas convection inside the reactor have shown that the size sorting effect arises from strong temperature gradients in the vertical direction that the gas convection produces. These results create new opportunities towards the production of high-quality low-dispersion silicon microsphers in gas phase by CVD.

## Additional Information

**How to cite this article**: Garín, M. *et al*. *In situ* size sorting in CVD synthesis of Si microspheres. *Sci. Rep.* **6**, 38719; doi: 10.1038/srep38719 (2016).

**Publisher's note:** Springer Nature remains neutral with regard to jurisdictional claims in published maps and institutional affiliations.

## Figures and Tables

**Table 1:** Values of the main parameters used for the calculation of the gravitational and thermophoretic forces acting on the particles.

Parameter	Value
ρ_p_	2.33 g·cm^**−**3^
ρ_g_	2.54 × 10^−4^ g·cm^−3^
μ	9.58 × 10^−6^ Pa·s
*k*_p_	149 W·m^−1^·K^−1^
*k*_g_	16.34 × 10^−3^ W·m^−1^·K^−1^
*C*_m_	1.14
*C*_s_	1.17
*C*_t_	2.18
λ	0.46 μm

The gas density has been calculated according to the pressure and the temperature at the reaction conditions, which are around 0.23 atm and 420 °C respectively for an initial condition of pressure of 0.1 atm at room temperature.
